# A Role of Eye Vergence in Covert Attention

**DOI:** 10.1371/journal.pone.0052955

**Published:** 2013-01-31

**Authors:** Maria Solé Puig, Laura Pérez Zapata, J. Antonio Aznar-Casanova, Hans Supèr

**Affiliations:** 1 Department Basic Psychology, Faculty of Psychology, University of Barcelona, Barcelona, Spain; 2 Institute for Brain, Cognition and Behavior, University of Barcelona, Barcelona, Spain; 3 Catalan Institution for Research and Advanced Studies, Barcelona, Spain; Barrow Neurological Institute, United States of America

## Abstract

Covert spatial attention produces biases in perceptual and neural responses in the absence of overt orienting movements. The neural mechanism that gives rise to these effects is poorly understood. Here we report the relation between fixational eye movements, namely eye vergence, and covert attention. Visual stimuli modulate the angle of eye vergence as a function of their ability to capture attention. This illustrates the relation between eye vergence and bottom-up attention. In visual and auditory cue/no-cue paradigms, the angle of vergence is greater in the cue condition than in the no-cue condition. This shows a top-down attention component. In conclusion, observations reveal a close link between covert attention and modulation in eye vergence during eye fixation. Our study suggests a basis for the use of eye vergence as a tool for measuring attention and may provide new insights into attention and perceptual disorders.

## Introduction

**Figure 1:**
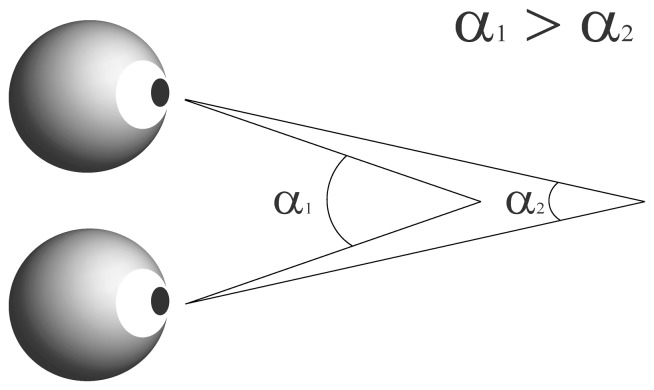
Schematic explanation of the angle of eye vergence. The eyes focus on a single point in space. The angle of eye vergence relates to the distance of the focus point to the eyes. For a near point the vergence angle (α1) is larger than for a far point (α2). α represents the angle of eye vergence.

Humans, like several other animals, have their two eyes positioned on the front of their heads, and provide us with a single visual field. The eyes receive a slightly different projection of the image because of the two eyes' different positions on the head. Therefore when looking at an object, the eyes must rotate around a vertical axis so that the projection of the image is in the center of the retina in both eyes. Vergence refers to the simultaneous movement of both eyes in opposite directions to obtain single binocular vision. The eyes rotate towards each other (convergence) when looking at an object closer by, while for an object farther away they rotate away from each other (divergence). Vergence is therefore an important cue in depth perception. The angle of vergence (AoEV) corresponds to the angle generated when both eyes focus on one point in space (Fig. 1).

Humans receive a surplus of sensory information. To cope with this, spatial attention is shifted to select relevant information at the expense of the rest. Usually, visuospatial attention moves about the environment in tandem with the eyes (overt attention). However, in the absence of overt orienting movements, attention also produces biases in perceptual and neural responses (covert attention; [1]–[3]). During eye fixation, small fixational eye movements (micro-saccades) relate to covert attention [4], [5], but see [6]. These findings corroborate the close connection of oculomotor system with visual attention.

Here we report another type of fixational eye movement, namely eye vergence that relates to covert attention. We show that during gaze fixation visual stimuli modulate the AoEV as a function of their ability to capture attention. Vergence angle increases after visual stimulation, and this enhancement correlates with bottom-up and top-down induced shifts in visuospatial attention. The start of the modulation in eye vergence is locked to the onset of the stimulus, while the size of the angle of eye vergence depends on the attentional load that the stimulus receives or attracts.

We argue that our observations have implications for theories of attention [7]–[14], and support a relationship between bottom-up and top-down attention, which are associated with segregated neuronal circuits [15], [16]. Finally, our study shows that there is a basis for using eye vergence as a tool for measuring attention, and may provide new insights into attention and perceptual disorders.

## Results

**Figure 2:**
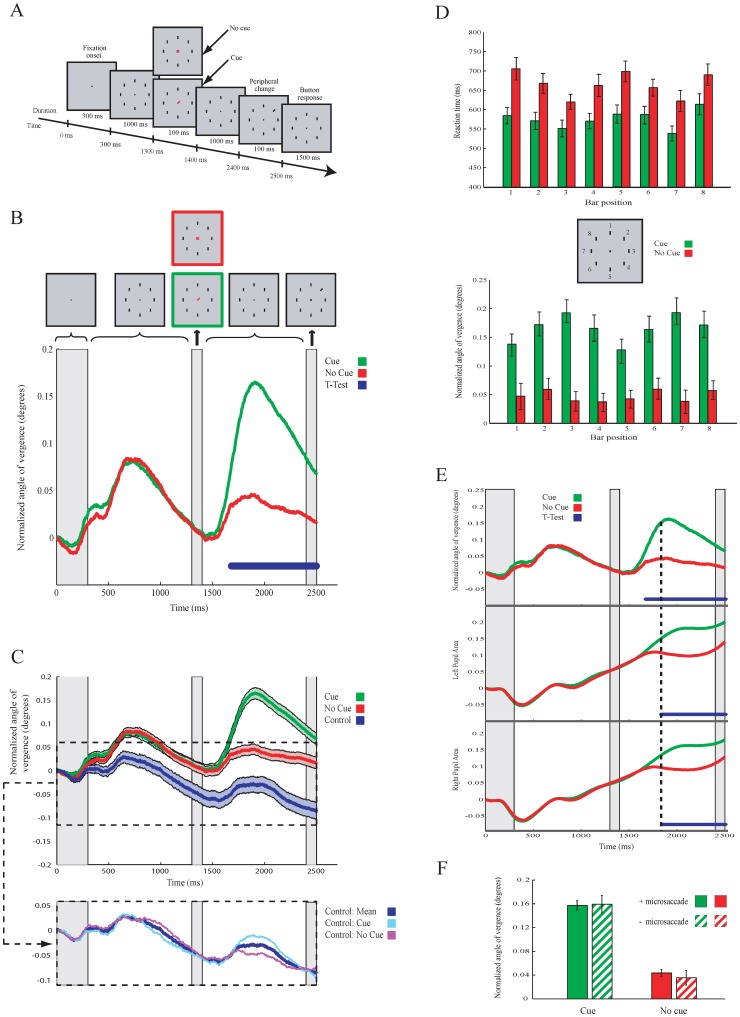
Visual search task of *Experiment 1* and modulation in eye vergence while performing the task. **A**. Illustration of the Cue/no-cue task. **B**. Average (across all subjects) size of AoEV in the cue (green) and no-cue (red) conditions over time. Time points (blue) indicate a significant difference in AoEV between both conditions. **C**. As in B, but with the average modulation in AoEV (blue trace) from a control task (*Experiment 2*). Shaded areas represent ±1 times SEM around the mean. Lower panel shows the modulation in AoEV separately for the cue and no-cue conditions of the control task. **D**. Mean reaction time and size of the AoEV for individual targets. Error bars are SEM. **E**. Modulation of AoEV (upper panel) and pupil size of the left (middle) and right (lower) eye. **F**. Mean sizes of AoEV in trials with and without micro-saccades in the cue and no-cue condition. Error bars are SEM.

We tested subjects in a visual cue/no-cue paradigm (*Experiment 1*) and measured the angle of eye vergence (AoEV). Once subjects had fixated on a central cross for 300 ms, 8 vertical bars ( =  possible targets) appeared around it (Fig. 2a). Subjects were given a valid cue (a small central line pointing to the target's position) in 50% of the trials, to inform them about the target location. In the other half of the trials, a no-cue stimulus (a central cross) was presented. Then one ( =  target) of the 8 vertical bars was titled (20°) for 100 ms and subjects had to identify the direction of the tilt. Faster reaction times (RT) were found in the cue condition than in the no-cue condition (mean ± sem RT: 587±8.2 ms vs. 688.8±9.3 ms, t-test_,_ p<0.01, df = 611). Detection performance was also slightly better when the target was cued (92.4% vs. 84.6%).

The positions of both eyes were simultaneously monitored during the task to compute the AoEV. Surprisingly, the size of the AoEV was not constant, but was affected by visual stimulation. Once a visual stimulus had been presented (i.e. the presentation of the central fixation spot, the array of vertical bars and the cue/no-cue stimulus, see Fig. 2a), the AoEV transiently increased with a mean velocity of 0.47±0.06°/s (Fig. 2b). To find out whether the fluctuations in the AoEV relate to shifts of attention, we compared for all time samples the average strength of the modulation in AoEV for the cue and no-cue conditions. We found that the modulation of the AoEV was significantly (t-test, p<0.01, df = 611) greater after cue onset (Fig. 2b, blue points). This difference became significant at 290 ms. The maximum of the average (non-normalized) AoEV was 350% higher in the cue condition than in the no-cue condition (mean ± sem: maximum of 0.12°±0.0068 at 612 ms versus 0.035°±0.0066 at 574 ms). The greater increase in the size of the AoEV in the cue condition was found for all target positions (Fig. 2d), although it was slightly higher for the horizontally located targets. As the increase in the AoEV occurred for all target locations, the observed effects do not reflect the nature of eye vergence (i.e. horizontal eye movements).

To support the idea that the greater increase in the AoEV after cue onset represents a cognitive mechanism, we analyzed the AoEV in subjects who viewed the same visual stimulation sequence as in Experiment 1, but without any instructed task (Experiment 2). The results show similar modulated responses in the AoEV over time, although the baseline was lower (Fig. 2c, upper panel). However, the modulation of the AoEV was as strong (t-test, for all time samples p>0.28, df = 160) after the cue stimulus as after the no-cue stimulus (Fig. 2c, lower panel). This demonstrates that the cue-induced change in the AoEV depends on the subject's engagement in the task.

**Figure 3:**
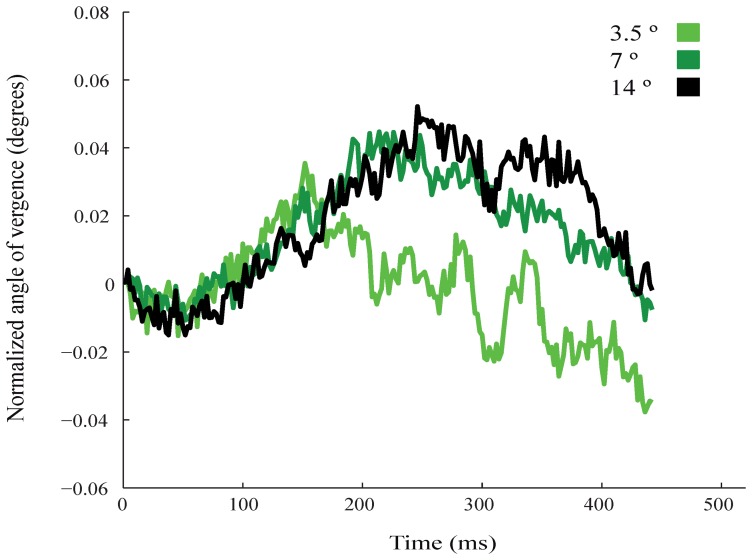
Control task showing modulation in eye vergence for targets located at different eccentricities from the fixation point. Data is from the cue condition. Colors denote eccentricity. Time is 320 ms from cue onset.

Pupil size, which relates to attention [17]–[19], might influence the measurement of the AoEV. We therefore analyzed pupil size. The results show that the size of the pupil increased during the trial more after cue onset than after no-cue onset (Fig. 2e). The difference in pupil size between the cue and no-cue conditions occurred at 572 ms (p<0.01, t-test, df = 611, blue dots in Fig. 2e), which is ∼200 ms later than the cue vs no-cue difference in AoEV (vertical dotted line in Fig. 2e). This supports the proposed relation of pupil size with shifts in visuospatial attention. We also tested for micro-saccades, which relate to attentional shifts [4], [5] but see [6]. Our results show that the differential modulation of the AoEV in cue and no-cue conditions is independent of the occurrence of micro-saccades (Fig. 2f). Furthermore we tested the effects of target eccentricity on the modulation of vergence. Targets were located at 3.5^0^, 7^0^, and 14^0^ from the fixation cross. As before, vergence angle increased after cue onset. The increase appeared to be weaker for targets at 3.5^0^ than for more peripheral targets. However, no significant differences in the modulation strength between all conditions was observed (t-test for all time samples; 3.5° vs 7° df = 172, 3.5° vs 14° df = 187, 7° vs 14° df = 181; for all p>0.05; Fig. 3).

**Figure 4:**
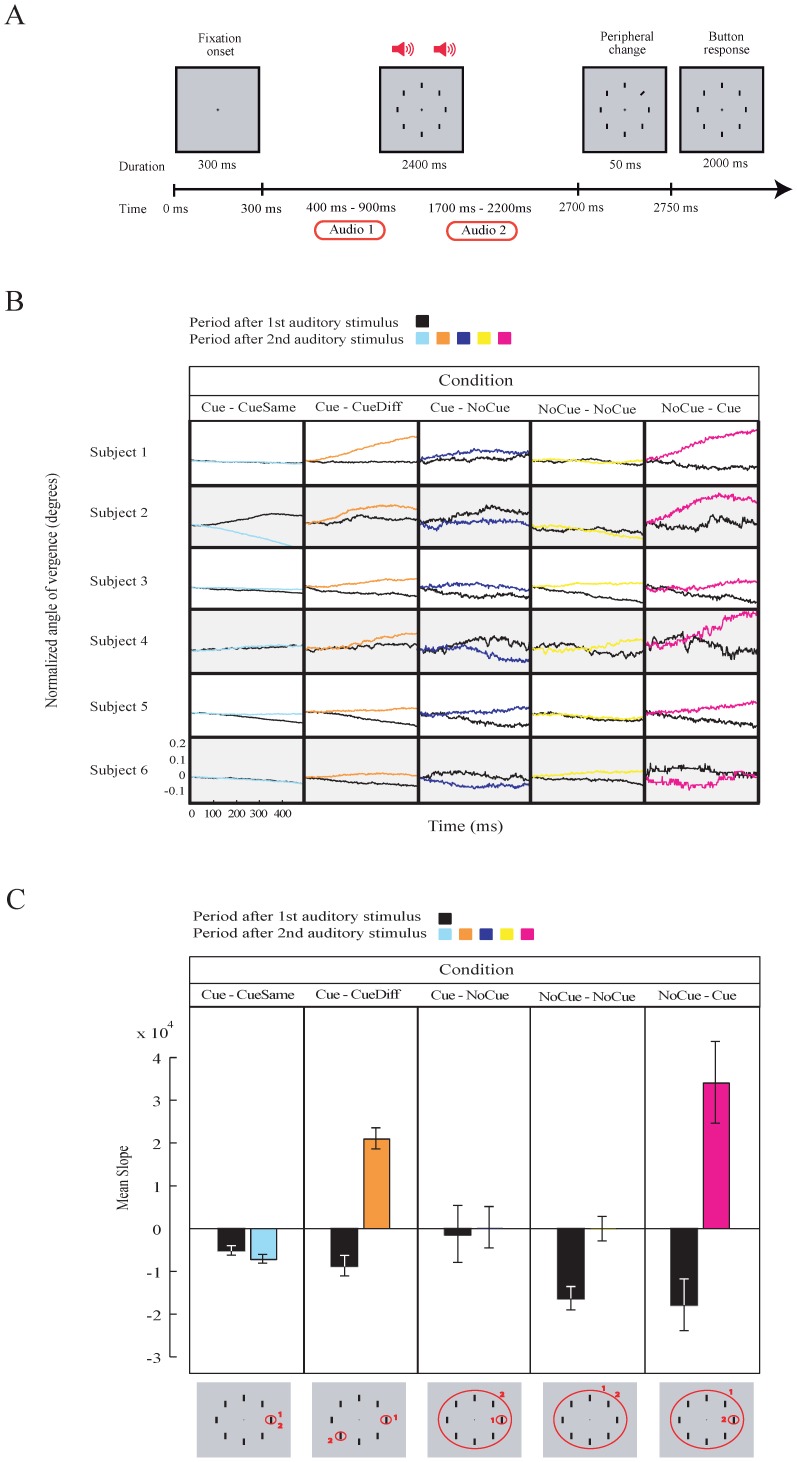
Visual search task combined with auditory cues (***Experiment 3***) **and modulation in eye vergence.** **A**. Illustration of the auditory task. Symbols denote cue. Two consecutive cues are given to the subjects **B**. Average size of AoEV after the onset of the 1^st^ (black traces) and 2^nd^ (colored traces) auditory cue for individual subjects. **C**. Comparison between the slopes of the modulation (taken from windows in B) of AoEV after the 1^st^ and 2^nd^ auditory cue. Grey panels below illustrate the shift in visuospatial attention (red circles) for each condition. Small circles indicate focused attention to a single target while a large circle indicates global or more spread attention to all possible target location. Numbers indicate the size and position of the attention window after the 1^st^ (1) and 2^nd^ (2) cue. Error bars are SEM.

A possible confounding factor is that the cue and no-cue stimuli are slightly different, which could produce a differential effect on fixational eye movements; especially as cue/no-cue stimuli were presented at the fovea. To exclude this effect of foveal stimulation, and to provide further evidence for the role of eye vergence in shifts of visuospatial attention, we conducted a visual discrimination experiment (*Experiment 3*) with two succeeding auditory cues. Auditory cues (pronounced in their native tongue) were numbers from 0 (NoCue) to 8 (1–8, numbers indicated one of the eight possible peripheral positions; Fig. 4a). Participants could practice until they correctly associated the numbers with the stimulus positions. In 89% of the trials, the first cue was given and was either valid (80%) or invalid (20%). In the other trials (11%), a no-cue was presented. The second stimulus was a valid cue in 89% and a no-cue in 11% of the trials. Therefore, there were five conditions: 1) Cue→CueSame, 2) Cue→CueDiff, 3) Cue→NoCue, 4) NoCue→NoCue, 5) NoCue→Cue. Overall performance correct was 91%. Reaction times were (mean ± sem): Cue→CueSame, 487±5.14 ms; Cue→CueDiff, 520±13.73 ms; Cue→NoCue, 606±32.55 ms; NoCue→NoCue 579±19.25 ms; NoCue→Cue, 479±27.23 ms. In the Cue→CueDiff (i.e. the first cue is an invalid cue) and NoCue→Cue conditions, we expect a shift of visuospatial attention after the second cue. In the Cue→CueSame and NoCue→NoCue, attention is not shifted after the second cue. In the Cue→NoCue, no shift of attention to a particular local region is required, as in the no-cue condition in *Experiment 1*. This interpretation is depicted in the lower panel of figure 4c.

The eye recording results show that initially after cue presentation the AoEV decreases during the period. This is because previously the AoEV had increased by the presentation of the 8 possible targets and at the time of the cue stimulus eye vergence was still was returning towards baseline levels. However, compared to the decrease after no-cue condition, the decrease in the cue condition is much smaller (black traces in Fig. 4b, and compare the first three black bars to the last two in Fig. 4c). The difference in AoEV between cue and no-cue condition was less pronounced, probably because of the different task and cue types (auditory versus visual) that were used. After the presentation of the second auditory cue, when attention is expected to shift in the Cue→CueDiff and NoCue→Cue condition, we observed a clear increase in the size of the AoEV (Fig. 4b,c; Table 1). In contrast, no increase in the size of the AoEV was noticed in the Cue→CueSame and Cue→NoCue conditions. In the NoCue→NoCue condition, a difference in AoEV was observed because of the lack of modulation of the AoEV after the first no-cue stimulus (the AoEV continued to decrease as a consequence of the increase in the AoEV that resulted from the previous presentation of the array of vertical bars; see also Fig. 2b) and the second no-cue stimulus. These findings demonstrate that when an auditory cue shifts visuospatial attention to a new target, the AoEV increases.

**Table 1:** Squared R values of the linear regression lines fitted by least square method to the data samples of the windows after the 1st and 2nd cue for the different conditions of experiment 2.

	Cue-CueSame	Cue-CueDIff	Cue-NoCue	NoCue-NoCue	NoCue-Cue
**1^st^ Cue**	*0.93*	*0.97*	*0.079*	*0.98*	*0.954*
**2^nd^ Cue**	*0.98*	*0.98*	*0.002*	*0.007*	*0.98*

**Figure 5:**
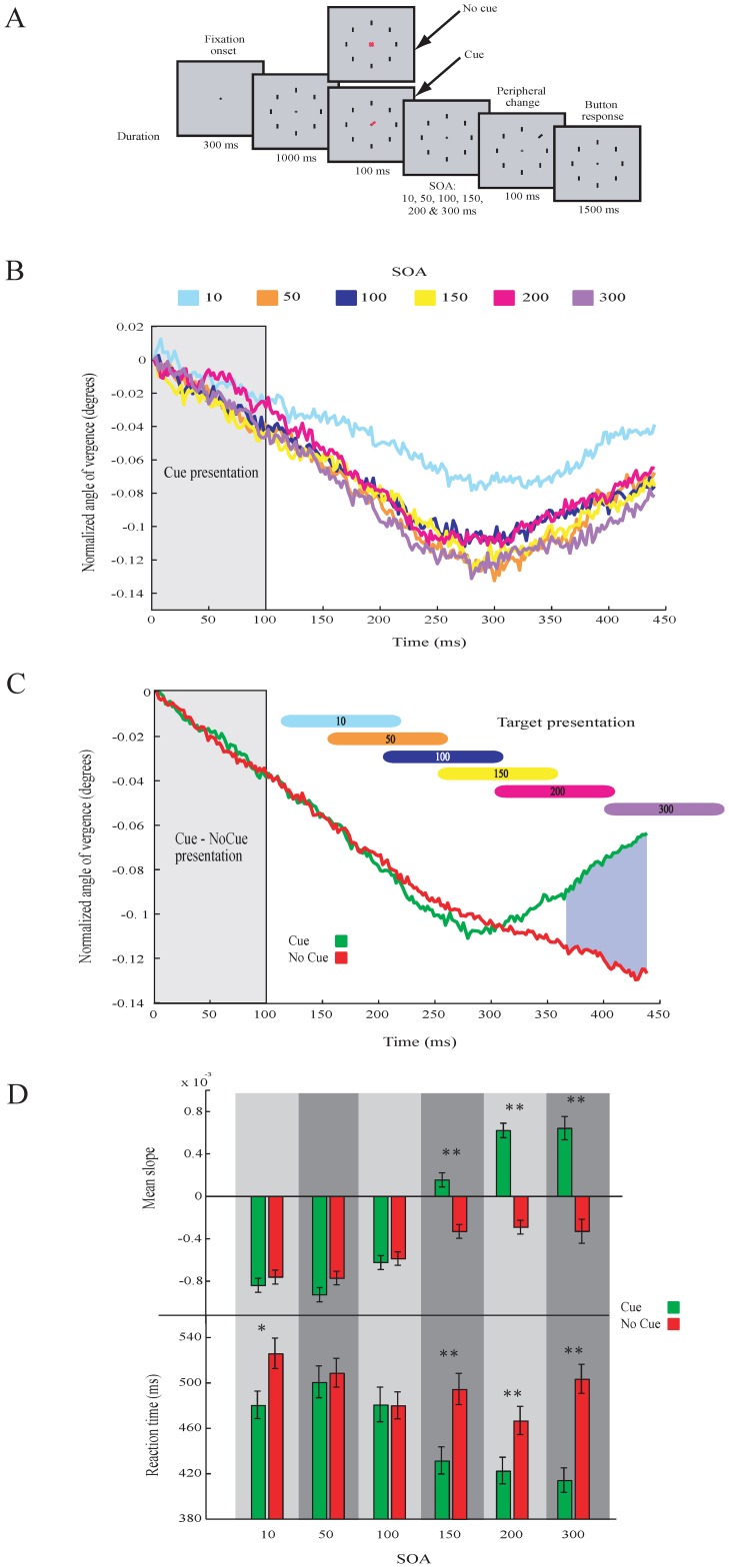
Visual search task (***Experiment 4***) **with different SOA and modulation in eye vergcence.** **A**. Illustration of the task. **B**. Average modulation across all subjects in AoEV separately for the different conditions (SOA). **C**. Average modulation in AoEV across all conditions. Colored vertical bars indicate the window of target presentation. Blue shaded area denotes a significant (p<0.01) difference between the cue and no-cue condition. **D**. Slopes of the modulation of AoEV and mean reaction times for the cue and no-cue of the different conditions (SOA). Bars represent the mean slopes, calculated for each condition (windows of 100 ms after target onset). Asterisks denote significant (* = p<0.05, ** = p<0.01, t-test) differences. Error bars are SEM.

To better understand the relation between the AoEV and visuospatial shifts of attention, we tested subjects in a task (*Experiment 4*) that was identical to the first visual task (*Experiment 1*), except for that fact that the time (stimulus onset asynchronies, SOA) between cue removal and target onset varied (Fig. 5a). The SOAs used were 10, 50, 100, 150, 200 and 300 ms. Detection performance and reaction times (mean ± sem) were: SOA 10 ms: 87.6%, 503±9.1 ms; SOA 50 ms: 90.7%, 504.4±9.4 ms; SOA 100 ms: 91.2%, 480.1±9.7 ms; SOA 150 ms: 95.0%, 463.1±9.2 ms; SOA 200 ms: 94.9%, 443.3±8.5 ms; SOA 300 ms: 94.6%, 457.1±8.0 ms. The eye data show that the size of the AoEV starts to increase around 290 ms after cue onset in all conditions (Fig. 5b). This is in agreement with the onset found in *Experiment 1*. The increase in the AoEV is thus coupled to the onset of the cue and not to the time of the presentation of the target. We then compared the strength of the modulation of the AoEV in the cue and no-cue conditions (Fig. 5c). The comparison shows that the mean difference in the AoEV between the cue and no-cue conditions occurs for SOAs of 150 ms and longer, but not for shorter SOAs (Fig. 5d, Table 2; t-test, df = 3413, p<0.01 in conditions for SOA of 150, 200 and 300 ms). This result is mimicked by behavioral RT. The RT were similar for cue and no-cue trials with short SOAs (50 and 100 ms), but for longer SOAs (150, 200 and 300 ms) the RT was faster (t-test, df = 3413, p<0.05 in the condition SOA 10 ms; p<0.01 in conditions SOA 150, 200 and 300 ms; Fig. 5d). Hence, we consider that attention shifts around 250 ms (i.e. 100 ms target duration plus 150 ms SOA) after target onset and is accompanied by an increase in the AoEV. The SOA 10 ms shows some peculiarities. Here the modulation in eye vergence is present but the overall level is higher than the other SOA conditions (Fig. 5b). Also the behavioral reaction times for SOA 10 ms are different between the cue and no-cue condition (Fig. 5d).

**Table 2:** Squared R values of the linear regression lines fitted by least square method to the data samples of the windows after the cue and no-cue onset for the different SOA conditions of experiment 4.

	SOA 10ms	SOA 50ms	SOA 100ms	SOA 150ms	SOA 200ms	SOA 300ms
**Cue**	*0.99*	*0.99*	*0.92*	*0.20*	*0.96*	*0.98*
**No Cue**	*0.98*	*0.99*	*0.95*	*0.95*	*0.94*	*0.59*

**Figure 6:**
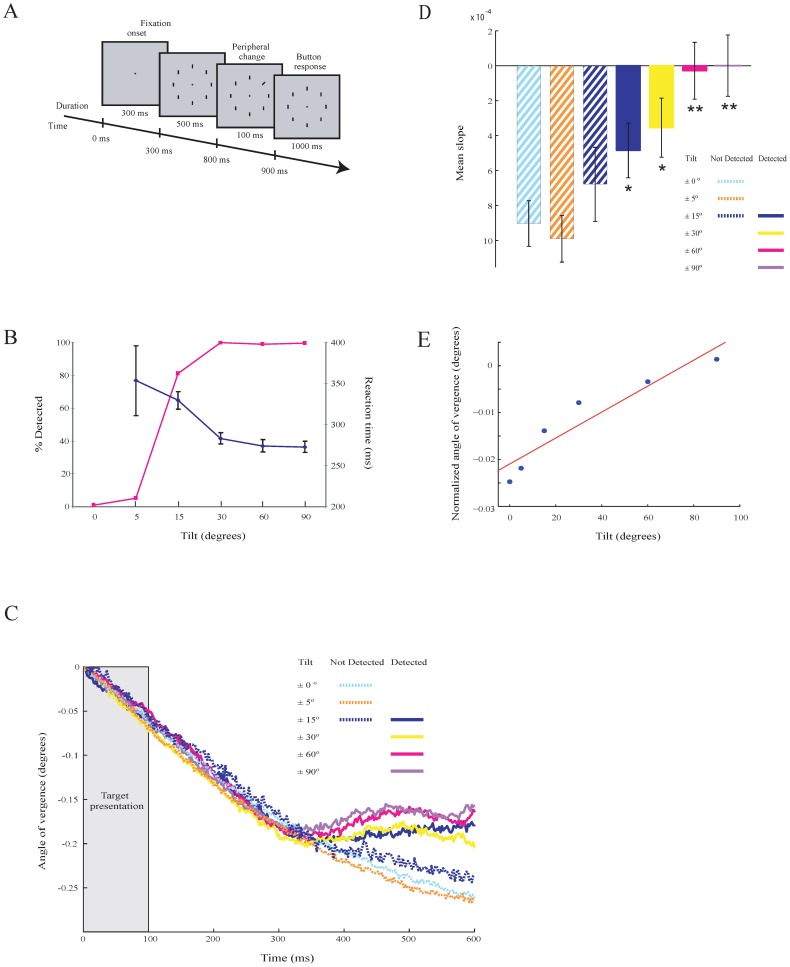
Detection task (***Experiment 5***) **and responses.** **A**. Illustration of the task. **B**. Detection performance (red) and reaction times (blue). Error bars are SEM. **C**. Modulation in AoEV of one subject separately for the different conditions (tilt) and behaviors (detected and undetected). **D**. Slopes of the modulation of the AoEV for the different conditions and behaviors. Asterisks denote significant (* = p<0.05, ** = p<0.01) differences (compared to condition 0^0^). Error bars are SEM. **E**. Mean vergence angle of selected window from all conditions plotted as a function of stimulus contrast. A linear regression line (red) is fitted.

To probe for a relation between the modulation of AoEV and bottom-up induced shifts in spatial attention we tested subjects in a detection task (*Experiment 5*). In this task (Fig. 6a), one of the vertical bars ( = target) was briefly tilted at various degrees (0° [no change], 5°, 15°, 30°, 60° and 90°), thereby modulating the saliency of the target as evidenced by performance (Fig. 6b). In the 0° case, attention will not be shifted to a particular place as there is no target. Detection performance and reaction times (mean ± sem) were: 5° = 5.1%, 353±42.5 ms; 15° = 81.3%, 330±10.7 ms; 30° = 100%, 283±6.8 ms; 60° = 99.1%, 274±7.4 ms and 90° = 99.6%, 273±6.7 ms. We then analyzed the strength of the modulation of AoEV after the stimulus onset. Again, the AoEV decreased because of the previous stimulation (see Fig. 2b), but around 300 ms after target onset, we observed an increase in the size of the AoEV.

This increase in the AoEV was a function of stimulus orientation (Fig. 6c–e; Table 3). It was most pronounced when the stimulus contrast was strongest and gradually decreased for lower contrast levels. The strength of the AoEV was positively correlated (R^2^ = 0.89) with stimulus contrast (Fig. 6e). In comparison to the 0° condition, we observed a significant difference in the size of AoEV (t-test, p<0.05 for the conditions 15^0^, df = 419 and 30^0^, df = 452; p<0.01 for the conditions 60^0^, df = 455, and 90^0^, df = 457). The difference in AoEV in the 15^0^ condition was however only significant for detected targets and not for undetected targets (Fig. 6d). For the lowest stimulus contrast (5°), which was below detection threshold (see Fig. 6b), the size of the AoEV did not significantly differ from the 0° (i.e. no change) condition (Fig. 6d). In conclusion, these observations show that the increase in the size of the AoEV correlates with visual detection and bottom-up induced visual attention.

**Table 3:** Squared R values of the linear regression lines fitted by least square method to the data samples of the windows after the cue and no-cue onset for the different stimulus contrasts of experiment 5.

	0°	5°	15°	30°	60°	90°
**Cue**	*0.97*	*0.98*	*0.87*	*0.70*	*0.023*	*0.00*

## Discussion

In this study, we report the relation between the angle of eye vergence (AoEV) and covert attention. We observed that the AoEV increases after the presentation of a cue or a peripheral stimulus while maintaining fixation at the center point. At first sight, a logical explanation for our results is to consider the distance of the peripheral target location. After the presentation of the cue subjects focus on the peripheral target (while maintaining fixation at the central point), which is slightly further away from the eyes than the central fixation point. The eyes then diverge to a more distant plane. In this situation the AoEV should decrease after cue presentation. However, opposite to such an expected reduction in AoEV we found an increase in vergence angle. In addition, the peak modulation in AoEV occurred well before target onset and at target onset when one would expect the subject to focus on the target, the AoEV decreased towards the initial values. Also, AoEV modulated after the onset of the fixation point (see Fig. 2b) when yet no peripheral targets were presented. Furthermore, in Cue-CueDiff condition of the third experiment attention shifted to different target locations. We observed that in this condition AoEV changed despite the identical distances of the targets to the fixation point. Therefore we believe that the distance to the target cannot explain the modulation in vergence. This conclusion is supported by the finding that target eccentricity did not correlate with vergence modulation. Also the results of the last experiment where vergence modulation correlates with stimulus contrast and perception indicates that distance is not the main explanatory factor. Moreover, our observed size in vergence modulation is about one factor lower than the expected vergence changes by depth. Finally, our findings indicate that the changes in pupil size does not cause the observed changes in AoEV as the temporal modulation in pupil size did not correspond to the temporal modulation in AoEV. For instance, pupil size showed a steady increase while modulation in vergence angle fluctuated over time (see Fig. 2e).

Instead, we speculate that vergence modulation reflects a shift in visual attention. As the change in the AoEV during fixation represents vertical eye movements, our results have consequences for the dissociation between eyes and covert attention [10], [14], [20] and for theories of attention [7]–[9], [11]–[13] in general. Based on the results from the first and last experiment, we also propose that eye vergence links bottom-up and top-down attentional mechanisms, which are believed to be associated with segregated neuronal circuits [15]. In line with our suggestion is recent evidence showing that the frontal cortex, where attention originates [10], [21]–[23], controls eye vergence [24]. In addition, a recent study that demonstrates that the frontal cortex is involved both in top-down attention and in bottom-up attention [25]. However, vergence modulation is observed for all target locations and is, unlike covert attention, not spatial specific. This means that the possible effect of vergence on sensory processing has to become selective for one single target location (otherwise in the no-cue condition vergence modulation should be strong as well!). So, further studies are needed to elucidate the possible role of eye vergence modulation in attention.

Disparity neurons in the primary visual cortex can detect the existence of disparity in their input from the eyes. These neurons are believed to provide depth information of the visual scene. Our current findings imply that retinal disparity changes during shifts of attention and accordingly the activity of disparity neurons. We therefore speculate that disparity neurons have besides a role in depth perception, also a role in attention and form part of the attention system in the brain. This suggestion is in accordance with findings of a disconnection of vergence movements and depth perception [26], [27] and with and unexpected specialization for horizontal disparity in primate primary visual cortex [28]. Moreover, recent evidence shows that ocular dominance maps may serve as a scaffold for the formation of disparity maps [29]. Ocular dominance columns in the visual cortex are formed during early ontogenetic stages and can be modified after birth during the sensitive period. In our study we provide indirect evidence for a connection between shifts of attention and disparity neurons. If true then our findings suggest that precise cortical developmental organization, i.e. correct axonal termination patterns and neuronal positioning into ocular columns, is beneficial for subsequent attentional processing of incoming sensory information [30].

## Materials and Methods

### Participants

The study was approved by the Ethics committee of the Faculty of Psychology of the University of Barcelona in accordance with the ethical standards laid down in the 1954 Declaration of Helsinki. We tested subject in several visual detection tasks (see Methods).

Twelve participants took part in Experiment 1 and Experiment 2 (1 man and 11 women, 22.9±1 age) from which 4 participated in the experiment with different target eccentricities. Six participants took part in Experiment 3 (1 man and 5 women, 25.3±1.6 age). Six participants performed Experiment 4 (1 man and 5 women, 25.3±1.6 age) and 4 participants (all women, 23.5±2.4 age) took part in Experiment 5. All participants had normal or corrected-to-normal vision. Participants received credits for courses or money for taking part in the experiment. We obtained written informed consent from all participants involved in our study.

### Apparatus

We used in-house C++ software and EventIDE (Okazolab Ltd, London, UK) for presenting the stimuli. The display resolution was 1024×768 pixels. The participants' position of gaze was monitored using a binocular EyeLink II eye-tracking system at 500 Hz (SR Research System, Ontario, Canada). To compensate for any head movements, we used individually molded bite bars (UHCOTECH Head Spot, University of Houston, Texas, USA).

### Procedure

Participants sat in a dimly lit (9 cd/m2) room, in front of the PC monitor at a distance of 47 cm. The eye tracking equipment was calibrated for each participant at the beginning of each set (standard 9 point calibration). Before starting the task, participants could practice with some training trials.

### Experiment 1. Visual cue/no-cue experiment

The experiment consisted of 4 sets with 32 trials each (128 trials in total). After eye calibration, observers were required to fixate a central cross (5×5 pixels). After 300 ms, 8 peripheral bars (3×11 pixels, eccentricity of 7.5°) appeared. In a separate control experiment of 2 sets we used eccentricities of 3.5°, 7.0° and 14°. After 1000 ms, a cue (a red line pointing to one of the peripheral positions, 3×13 pixels) or a no-cue (a red cross, 13×13 pixels) stimulus appeared for 100 ms in the central position. After an additional period of 1000 ms, one of the peripheral bars briefly (100 ms) changed its orientation (a tilt of 20° to the left or right). Participants had to respond by pressing a button as fast and accurately as possible to indicate whether the bar tilted to the left or to the right. Feedback was not given to the observers.

### Experiment 2. Visual experiment without task

In this experiment, the same subjects viewed the same visual stimuli sequence as in Experiment 1. However, the subjects were instructed to fixate the central cross without performing any task (1 set of 32 trials).

### Experiment 3. Auditory cue/no-cue experiment

The auditory experiment consisted of 360 trials. After eye calibration, observers were required to fixate a central cross (5×5 pixels). After 300 ms, 8 peripheral bars (eccentricity of 7.5°) appeared and 100 ms later, participants listened to an auditory stimulus (a number from 0 to 8 in Catalan). Each number (cue) indicated a peripheral bar position, except for number 0 (no-cue). The cues and no-cue were presented for 500 ms. After 800 ms, a second auditory cue was played, which was always valid. As before, it could be a number from 0 to 8. In 80% of cases, the first and the second auditory stimuli were the same. The percentage of trials for each condition was: 1) Cue-CueSame (71.1%), 2) Cue-CueDiff (15.6%), 3) Cue-NoCue (2.2%), 4) NoCue-NoCue (8.9%), 5) NoCue-Cue (2.2%). After 500 ms, one of the peripheral bars briefly changed its orientation (±20° for 50 ms). Participants had to indicate as fast and accurately as possible whether it tilted to the left or to the right. Feedback was not given to the observers.

### Experiment 4. Visual experiment with different delays (SOA)

This experiment was the same as Experiment 1 except that the time between cue onset and target onset randomly varied. The stimulus onset asynchronies (SOA) used were 10, 50, 100, 150, 200 and 300 ms. Subjects performed 384 trials (64 trials for each condition).

### Experiment 5. Visual contrast experiment

This experiment consisted of 384 trials (64 per condition). After eye calibration, observers were required to fixate a central cross (5×5 pixels). After 300 ms, 8 peripheral bars (eccentricity of 7.5°) appeared and 500 ms later, one of the peripheral bars briefly changed its orientation. The tilt could be to the left or to the right. We used 0° (no tilt), 5°, 15°, 30°, 60° and 90°. Participants had to respond by pressing a button as fast and accurately as possible if they detected the tilt.

### Data analysis

We calculated the angle of eye vergence by transforming the HRef recordings (X and Y coordinates of both eyes), provided by the Eye Link II software, into angular units through algorithms designed to calculate 3-D components of both eye gaze vectors. The transformation was performed taking into account the real distance of the screen to the observer and the actual inter-pupil distance. The AoEV is the point at which the intersection of both eye gaze vectors made the least error. For each subject, the eye vergence data were normalized by dividing the raw data by the maximum value of the recorded samples from fixation onset to target onset. Only correct trials were analyzed except in experiment 5. Detection of micro-saccades was done as described in [31].

For the calculation of the mean AoEV in Experiment 1 (including control experiments) and 2, we selected a window of 100 ms (1850 ms –1950 ms). This window was chosen because for all subjects it was centered on the maximum peak of AoEV after cue/no-cue onset. For the other experiments we selected per trial a time window and fitted a linear regression line by least square method through the sampled data points within a window. Windows were 500 ms/100 ms starting after audio offset/target onset in experiment 3/4. In experiment 5, a 100 ms window was taken 300 ms from target onset. These windows were chosen because for all subjects they coincided with the start of cue or target induced modulation in eye vergence, when visually inspected.

## References

[1] Posner MI (1980) Orienting of attention. The 7th Sir F.C. Bartlett Lecture. Quartly Journal Experimental Psychology 32: 3–25. 10.1080/00335558008248231 7367577

[2] Wright RD, Ward LM (2008) Orienting of Attention. Oxford University Press.

[3] Bisley JW (2011) The neural basis of visual attention. J Physiology 589: 49–57. 10.1113/jphysiol.2010.192666 PMC3039259 20807786

[4] Hafed ZM, Clark JJ (2002) Microsaccades as an overt measure of covert attention shifts. Vision Research 42: 2533–2545. 12445847 10.1016/s0042-6989(02)00263-8

[5] Engbert R, Kliegl R (2003) Microsaccades uncover the orientation of covert attention. Vision Research 43: 1035–1045. 12676246 10.1016/s0042-6989(03)00084-1

[6] Horowitz TS, Fine EM, Fencsik DE, Yurgenson S, Wolfe JM (2007) Fixational eye movements are not an index of covert attention. Psychol Sci, 18, 356. 10.1111/j.1467-9280.2007.01903.x 17470262

[7] LaBerge D (1983) Spatial extent of attention to letters and words. J Exp Psychol: Human Perception and Performance 9: 371–379. 10.1037//0096-1523.9.3.371 6223977

[8] Eriksen C, St James J (1986) Visual attention within and around the field of focal attention: A zoom lens model. Perception & Psychophysics 40: 225–240. 3786090 10.3758/bf03211502

[9] Desimone R, Duncan J (1995) Neural mechanisms of selective visual attention. Annu Rev Neurosci 18: 193–222. 7605061 10.1146/annurev.ne.18.030195.001205

[10] Corbetta M (1998) Frontoparietal cortical networks for directing attention and the eye to visual locations: Identical, independent, or overlapping neural systems? Proc Natl Acad Sci U S A 95: 831–838. 9448248 10.1073/pnas.95.3.831 PMC33805

[11] Yantis S, Serences JT (2003) Cortical mechanisms of space-based and object-based attentional control. Curr Opin Neurobiol 13: 187–193. 12744972 10.1016/s0959-4388(03)00033-3

[12] Hunt AR, Kingstone A (2003) Covert and overt voluntary attention: linked or independent? Cogn Brain Res 18: 102–105. 10.1016/j.cogbrainres.2003.08.006 14659502

[13] Maunsell JH, Treue S (2006) Feature-based attention in visual cortex. Trends Neurosci 29: 317–322. 16697058 10.1016/j.tins.2006.04.001

[14] Awh E, Armstrong KM, Moore T (2006) Visual and oculomotor selection: links, causes and implications for spatial attention. Trends Cogn Sci 10: 124–30. 16469523 10.1016/j.tics.2006.01.001

[15] Corbetta M, Akbudak E, Conturo TE, Snyder AZ, Ollinger JM, et al (1998) A common network of functional areas for attention and eye movements. Neuron 21: 761–773. 9808463 10.1016/s0896-6273(00)80593-0

[16] Corbetta M, Shulman GL (2002) Control of goal-directed and stimulus-driven attention in the brain. Nature Rev Neurosci 3: 201–15. 11994752 10.1038/nrn755

[17] Hoecks B, Levelt W (1993) Pupillary dilation as a measure of attention: A quantitative system analysis. Behavior Research Methods, Instruments & Computers 25: 16–26.

[18] Gabay S, Pertzov Y, Henik A (2011) Orienting of attention, pupil size, and the norepinephrine system. Attention, Perception & Psychophysics 73: 123–9. 10.3758/s13414-010-0015-4 21258914

[19] Wierda SM, van Rijnc H, Taatgen NA, Martens S (2012) Pupil dilation deconvolution reveals the dynamics of attention at high temporal resolution. Proc Natl Acad Sci U S A. doi:10.1073/pnas.1201858109. 10.1073/pnas.1201858109 PMC3365158 22586101

[20] Ignashchenkova A, Dicke PW, Haarmeier T, Their P (2004) Neuron-specific contribution of the superior colliculus to overt and covert shifts of attention. Nature Neurosci 7: 56–64. 14699418 10.1038/nn1169

[21] Kastner S, Ungerleider LG (2000) Mechanisms of visual attention in the human cortex. Annu Rev Neurosci 23: 315–341. 10845067 10.1146/annurev.neuro.23.1.315

[22] Moore T, Armstrong KM, Fallah M (2003) Visuomotor origins of covert spatial attention. Neuron 40: 671–683. 14622573 10.1016/s0896-6273(03)00716-5

[23] Thompson KG, Biscoe KL, Sato TR (2005) Neuronal basis of covert spatial attention in the frontal eye field. J Neurosci 25: 9479–87. 16221858 10.1523/JNEUROSCI.0741-05.2005 PMC2804969

[24] Gamlin PD, Yoon K (2000) An area for vergence eye movement in primate frontal cortex. Nature 407: 1003–1007. 11069179 10.1038/35039506

[25] Katsuki F, Constantinidis C (2012) Early involvement of prefrontal cortex in visual bottom-up attention. Nat Neurosci 15: 1160–1166. 22820465 10.1038/nn.3164 PMC3411913

[26] Masson GS, Busettini C, Miles FA (1997) Vergence eye movements in response to binocular disparity without depth perception. Nature 389: 283–286. 9305842 10.1038/38496

[27] Cumming BG, Parker AJ (1997) Responses of primary visual cortical neurons to binocular disparity without depth perception. Nature 389: 280–283. 9305841 10.1038/38487

[28] Cumming BG (2002) An unexpected specialization for horizontal disparity in primate V1. Nature 418: 633–636. 12167860 10.1038/nature00909

[29] Kara P, Boyd JD (2009) A micro-architecture for binocular disparity and ocular dominance in visual cortex. Nature 458: 627–631. 19158677 10.1038/nature07721 PMC2700034

[30] Leigh RJ, Zee DS (2006) The Neurology of Eye Movements. Oxford University Press, USA, pages 776.

[31] Supèr H, van der Togt C, Spekreijse H, Lamme VA (2004) Correspondence of presaccadic activity in the monkey primary visual cortex with saccadic eye movements. Proc Natl Acad Sci U S A 101: 3230–3235. 14970334 10.1073/pnas.0400433101 PMC365772

